# Amiodarone vs. metoprolol succinate in HFrEF complicated with persistent atrial fibrillation with rapid ventricular response: A prospective observational study

**DOI:** 10.3389/fcvm.2022.1029012

**Published:** 2023-01-09

**Authors:** Yongrong Liu, Yali Hong

**Affiliations:** Department of Cardiovascular Medicine, People's Hospital of Chongqing Hechuan, Chongqing, China

**Keywords:** amiodarone, metoprolol succinate, heart failure with reduced ejection fraction, atrial fibrillation, prospective observational study

## Abstract

**Background:**

β-blockers have been recommended for patients with heart failure (HF) and atrial fibrillation (AF), but studies have shown that β-blockers do not reduce all-cause mortality or cardiovascular mortality in patients with HF and AF.

**Objective:**

To investigate the difference in efficacy between oral amiodarone and metoprolol succinate for patients with HF with reduced ejection fraction (HFrEF) and persistent atrial fibrillation (pAF) with rapid ventricular response (RVR).

**Methods:**

Patients with HFrEF complicated with pAF with RVR treated in the People's Hospital of Chongqing Hechuan between March 2018 and March 2019 were enrolled in this prospective observational study. The primary outcomes were cardiovascular mortality and the first hospitalization for HF rate. The secondary outcomes were type B pro-brain natriuretic peptide (NT-proBNP) before/after treatment, left ventricular ejection fraction (LVEF) before/after treatment, average heart rate (AhR), and the rate of sinus rhythm after 1 year of follow-up.

**Results:**

A total of 242 patients with HFrEF complicated with pAF with RVR were enrolled and divided into amiodarone + perindopril + spironolactone+ routine drug (amiodarone group, *n* = 121) and metoprolol succinate + perindopril + spironolactone +routine drug (metoprolol succinate group, *n* = 121) according to their treatment strategy. Cardiovascular mortality (4.9 vs. 12.4%, HR: 2.500, 95%CI: 1.002–6.237, *P* = 0.040) and first hospitalization for HF (52.9 vs. 67.8%, HR: 1.281, 95%CI: 1.033–1.589, *P* = 0.024) were significantly lower in the amiodarone group than in the metoprolol group. The mean ventricular rate in the amiodarone group was significantly lower than in the metoprolol group (64.5 ± 3.2 vs. 72.4 ± 4.2, *P* < 0.001). After 1 year of follow-up, the sinus rhythm rate was significantly higher in the amiodarone group than in the metoprolol group (38.8 vs. 7.4%, HR: 0.191, 95%CI: 0.098–0.374, *P* < 0.001). The difference in proBNP (3,914.88 vs. 2,558.07, *P* < 0.001) and LVEF (−6.89 vs. −0.98, *P* < 0.001) before and after treatment was significantly higher in the amiodarone group than in the metoprolol group.

**Conclusion:**

In conclusion, in this prospective observational study, the amiodarone group had lower risk of cardiovascular death and the first hospitalization for HF than metoprolol in HFrEF and persistent atrial fibrillation (pAF) with RVR. The mechanism may be related to improved cardiac function, rhythm control and ventricular rate control.

**Registration number:**

ChiCTR2200057816; Registered 7 March 2022–Retrospectively registered: http://www.medresman.org.cn/pub/cn/proj/projectshshow.aspx?proj=4222.

## Introduction

According to existing literature, β-blockers such as bisoprolol, carvedilol, or metoprolol succinate can reduce cardiovascular mortality and sudden cardiac death in patients with heart failure (HF), reduce hospitalization for HF, and reduce all-cause mortality (1). Therefore, β-blockers remain the cornerstone of HF treatment (2–4). Although major guidelines do not recommend the use of specific β-blocker (1, 5), due to their effectiveness, metoprolol succinate is indicated for patients with HF (6). Still, only about 12% of patients with chronic HF in the stable phase reach the recommended target dose of β-receptor blocker, and about 17% reach the target heart rate (7). The negative inotropic effects of β-receptor blockers are related to the used doses, especially in patients with HF with reduced ejection fraction (HFrEF) and atrial fibrillation (AF) (8–10). Therefore, the titration of β-blockers to achieve the target dose or target heart rate to reduce mortality in HF patients remains difficult (8–10).

Amiodarone can control the sinus rhythm in a number of patients (11). In addition, amiodarone is a treatment option for AF patients who recover and maintain sinus rhythm (12). It can also be used to control ventricular rate (13). Compared with metoprolol succinate, amiodarone can exert its biological effect without titration (12, 14, 15). Metoprolol succinate can be used to manage AF with rapid ventricular rate response (RVR) in patients with HFrEF (16). Compared with the β-receptor blocker in the traditional therapy plan, amiodarone has a better ability to restore and maintain sinus rhythm in patients with HFrEF and AF (14, 17, 18). In addition, maintaining the sinus rhythm in patients with HFrEF and AF can improve patients' outcomes (19, 20).

The most difficult decision for the treatment of patients with HF and AF is to select either a ventricular rate control strategy or a rhythm control strategy. A previous randomized controlled study has shown no difference between a drug for rhythm control and a drug for ventricular rate control in clinical outcomes, and the rhythm-control strategy did not offer more survival advantages (21). Nevertheless, there are some patients who appear to benefit from rhythm control, including those with significant symptoms of atrial fibrillation (such as those with complete ventricular rate control but with persistent symptoms) and those with reversible cardiomyopathy (such as arrhythmia-induced cardiomyopathy) (19, 20). Therefore, this study aimed to compare the difference between the rhythm control strategy represented as oral amiodarone and the ventricular rate control strategy represented by metoprolol on cardiovascular death and the first hospitalization for HF in patients with HFrEF and persistent atrial fibrillation (pAF) with RVR.

## Methods

### Study design and participants

Patients with HFrEF complicated with pAF with RVR treated in the Department of Cardiology of People's Hospital of Chongqing Hechuan between March 2018 and March 2019 were enrolled in this prospective observational study. The study was registered in the Chinese Clinical Trial Registry (www.chictr.org.cn/) database (ChiCTR2200057816). It was approved by the ethics committee of People's Hospital of Chongqing Hechuan. Each enrolled participant signed the informed consent form. The data were analyzed from June 2021 to December 2021.

The inclusion criteria were: (1) 55–75 years of age; (2) electrocardiogram on admission showed AF with RVR (heart rate >100 bpm); (3) mean ventricular rate >100 bpm indicated by ambulatory electrocardiogram after drug therapy to dry body weight; (4) the inner diameter of left atrium >45 mm; (5) AF lasting for more than 1 month but < 1 year; (6) HFrEF and proBNP >1,200 pg/mL; (7) temporary discontinuation of metoprolol succinate due to New York Heart Association (NYHA) classification class IV (22).

The exclusion criteria were: (1) valvular heart disease requiring surgical intervention; (2) hyperthyroidism, severe hypothyroidism, or diagnosed hyperthyroid heart disease; (3) contraindications to amiodarone; (4) severe hepatic or renal insufficiency; (5) severe intraventricular block or complete left bundle branch block; (6) with atrial thrombosis confirmed by esophageal echocardiography; (7) AF ablation planned was performed within 1 year; (8) severe hypotension; (9) severe bradycardia.

### Diagnosis of HFrEF and persistent rapid AF

The HFrEF was diagnosed through echocardiography, and the left ventricular ejection fraction (LVEF) measured ≤ 40% (measured by Simpson's method), with typical symptoms and signs of HF, and proBNP >1,200 pg/mL when having AF. AF was diagnosed by the 12-lead electrocardiogram, which was recorded as AF with RVR and lasted more than 7 days.

### Echocardiography

For the evaluation of the cardiac systolic function in HFrEF, the left ventricular end-diastolic and end-systolic areas were measured at the level of the mitral valve and papillary muscles by using Simpson's method through transthoracic echocardiography (Philips XinYue IE33). Left ventricular end-diastolic and end-systolic lengths were measured in apical four-chamber sections and input into the software to calculate LVEF before and after treatment.

### 24-h ambulatory electrocardiogram

The 24-h ambulatory electrocardiogram (AECG) method allows the recording and monitoring of the ECG changes in the active and quiet states of the human heart over a long time. Each AECG report was considered valid if it was analyzed >80% of the valid recording time. The mean ventricular rate values were obtained from valid AECG reports. Conversion to sinus rhythm was considered if sinus rhythm was indicated to be without paroxysmal or persistent atrial fibrillation on the basis of AECG results after 1 year.

### Procedure

After admission, inpatients with HF with rapid pAF were initially screened by the attending physician based on inclusion and exclusion criteria. All participants required temporary discontinuation of metoprolol succinate due to NYHA class IV but continued the original doses of perindopril and spironolactone. The patients were divided into the amiodarone + routine drug group (amiodarone group, *n* = 121) and metoprolol succinate +routine drug group (metoprolol succinate group, *n* = 121) according to their treatment strategy. Routine drug included perindopril, spironolactone, diuretic, digoxin, rivaroxaban and etc.

### Medication during hospitalization

The participants in the amiodarone group were treated with oral amiodarone at 600 mg/d for the first week, 400 mg/d for the second week, and 200 mg/d for the third week combined with routine treatment. Since amiodarone has a certain cardioversion effect, anticoagulation for at least 3 weeks or esophageal echocardiography is required to exclude atrial thrombus before amiodarone is administered. The patients in the metoprolol succinate group were treated with oral metoprolol succinate starting gradually from 23.75 mg to the patient's maximum tolerated dose, combined with routinel treatment upon the improvement of the cardiac function. Routine treatment during hospitalization included furosemide and deslanoside for injection, furosemide tablets, potassium chloride sustained-release tablets, perindopril tablets, spironolactone tablets, rivaroxaban tablets, and digoxin tablets.

### Medication during outpatient follow-up

During the outpatient follow-up period, both groups were given digoxin tablets 0.125 mg/d, furosemide tablets 40 mg/d, spironolactone 20 mg/d, potassium chloride sustained-release tablets 1 g bid, perindopril tablets gradually titrated to the maximum tolerated dose, and rivaroxaban tablets 20 mg/d. The participants in the amiodarone group were given amiodarone tablets 200 mg/d. In addition, the participants in the metoprolol succinate group were treated with metoprolol succinate tablets starting from 23.75 mg. All the participants underwent a general ECG and blood pressure examination at the outpatient clinic every 2 weeks. According to the changes in heart rate, blood pressure, and NYHA classification of cardiac function, the perindopril and metoprolol succinate was gradually titrated to the maximum tolerated dose.

### Data collection

During follow-up, all participants underwent a general ECG and blood pressure examination every 2 weeks and a reexamining proBNP, cardiac color doppler ultrasound, and dynamic electrocardiogram every 3 months. In the amiodarone group, chest X-rays, liver function, and thyroid function were also checked every 3 months. Monthly telephone follow-up was conducted to collect data on cardiovascular deaths and hospitalizations of patients with HFrEF complicated with pAF with RVR. After 1 year of follow-up, all participants were routinely reexamined by dynamic electrocardiography to assess the proportion of conversion to sinus rhythm.

### Bias control

Since amiodarone can restore the sinus rhythm, in order to further reduce the risk of embolism caused by the conversion of AF in some patients during follow-up, all amiodarone group participants underwent transesophageal echocardiography, or rivaroxaban anticoagulant treatment for more than 3 weeks before enrollment. After that, they were treated with long-term rivaroxaban for anticoagulation. Since the LVEF of all participants was < 40%, most participants required long-term use of furosemide to improve their symptoms. In order to prevent hypokalemia caused by long-term use of diuretics and increase of proarrhythmic risk of amiodarone, during the outpatient and inpatient follow-up, potassium chloride sustained-release tablets were used to prevent hypokalemia.

### Outcomes

The primary outcomes were cardiovascular mortality and the first hospitalization for HF rates during follow-up. The secondary outcomes were proBNP before and after treatment (measured by the Getein1100 fluorescence immunoquantitative analyzer), LVEF before and after treatment (measured by the Simpson method), AhR, and the rate of sinus rhythm after 1 year of follow-up.

### Statistical analysis

Continuous data with normal distribution were presented as mean ± standard deviation (SD) and analyzed using Student's *t*-test or one-way analysis of covariance. Categorical data were expressed as n (%) and analyzed using the Chi-square test. Binary logistic regression was used for multivariable analysis to explore the influencing factors for cardiovascular mortality and first admission due to HF. Data were analyzed using SAS 9.0 (SAS Institute, Cary, NC, USA). Two-sided *P* < 0.05 were considered statistically significant.

## Results

**Figure 1:**
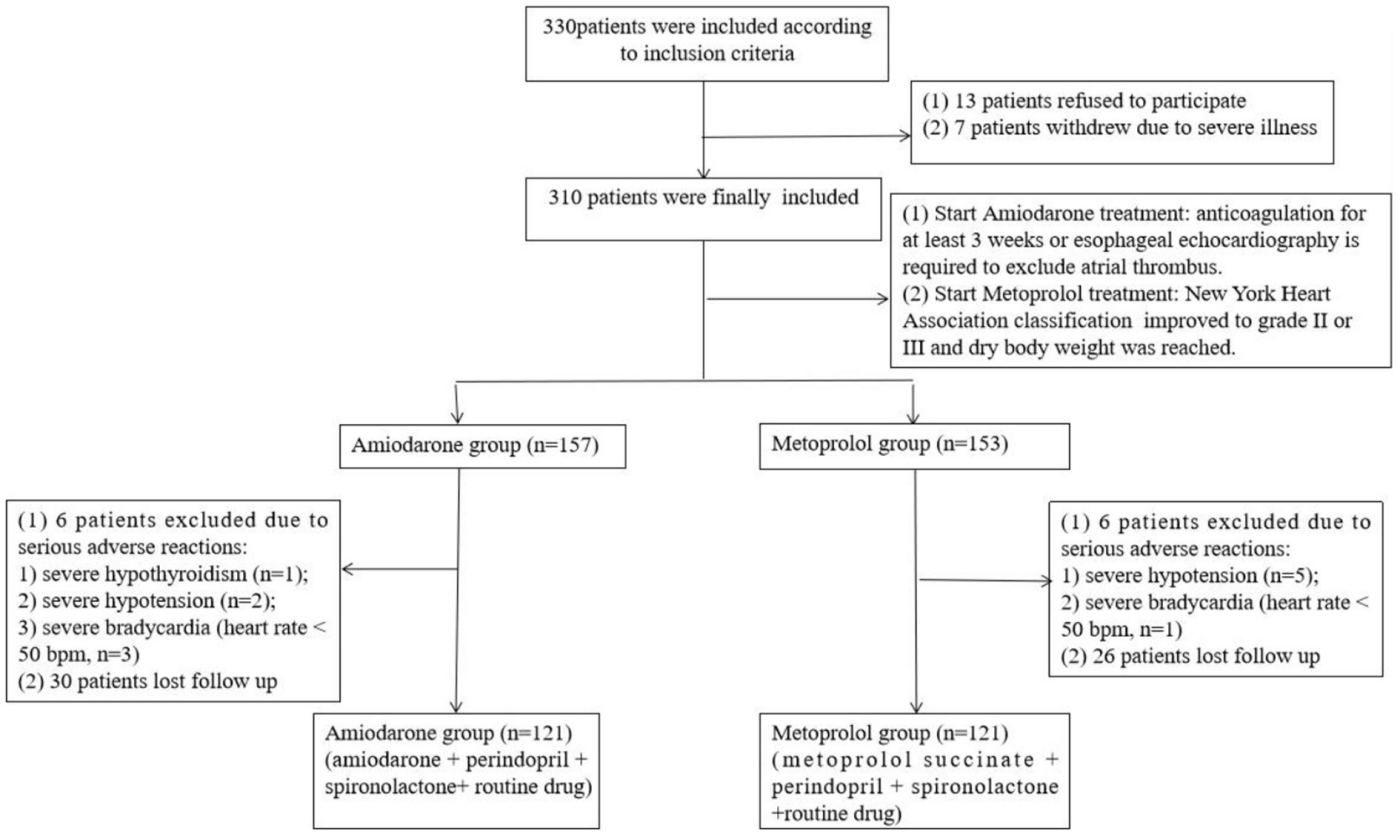
Participants' flowchart.

A total of 330 eligible patients were initially included in the study. Thirteen patients refused to participate, 7 patients chose to withdraw because of severe illness, 310 patients were included and divided into amiodarone and metoprolol succinate groups. In amiodarone group, 1 case of severe hypothyroidism, 2 cases of hypothyroidism and 3 cases of severe bradycardia were withdrew, and 30 cases lost follow up. In metoprolol succinate group, 5 cases of severe hypotension, 1 case of severe bradycardia were withdrew, and 26 cases lost follow up. The amiodarone and metoprolol succinate groups finally included 121 patients, respectively (Figure 1).

The sensitivity analysis found that the results of baseline characteristics between the two groups were basically the same in the full participants and after the follow-up population, suggesting that lost follow-up was randomly distributed between the two groups and had no effect on the results (Table 1).

**Table 1:** Characteristics of the participants.

	**Amiodarone (*n* = 151)**	**Metoprolol succinate (*n* = 147)**	***P***	**Amiodarone (*n* = 121)**	**Metoprolol succinate (*n* = 121)**	***P***
Male, *n* (%)	73 (48.3%)	75 (51%)	0.728	63 (52.1)	62 (51.2)	0.898
Age (years)	62.43 ± 4.70	60.98 ± 5.33	0.013	62.4 ± 4.7	61.2 ± 5.4	0.066
Diabetes, *n* (%)	42 (27.8%)	51 (34.7%)	0.213	37 (30.6)	41 (33.9)	0.582
Hypertension, *n* (%)	81 (53.6%)	92 (62.6%)	0.128	71 (58.7)	74 (61.2)	0.694
Serum creatinine (μmol/L)	77.27 ± 5.53	79.2 ± 6.15	0.005	77.42 ± 5.81	79.34 ± 6.53	0.016
Smoking history, *n* (%)	50 (33.1%)	49 (33.3%)	1.000	42 (34.7)	44 (36.4)	0.788
Digoxin, *n* (%)	135 (89.4%)	130 (88.4%)	0.855	114 (94.2)	112 (92.6)	0.605
Furosemide, *n* (%)	151 (100%)	147 (100%)	-	121 (100.0)	121 (100.0)	-
Spironolactone, *n* (%)	151 (100%)	147 (100%)	-	121 (100.0)	121 (100.0)	-
Dosage of perindopril, mg	12.79 ± 2.01	12.7 ± 2.30	0.728	12.34 ± 2.2	12.42 ± 2.4	0.787
Rivaroxaban, *n* (%)	151 (100%)	147 (100%)	-	121 (100)	121 (100)	-
Diameter of the left atrium, mm	53.34 ± 2.05	53.45 ± 2.23	0.654	53.4 ± 2.1	53.7 ± 2.4	0.543
Duration of AF, month	5.29 ± 2.59	5.23 ± 2.39	0.818	5.3 ± 2.7	5.1 ± 2.5	0.438
Types of cardiomyopathy, *n* (%)	64.46 ± 3.21	72.29 ± 4.32	1.000			0.794
Dilated cardiomyopathy	87 (57.6%)	84 (57.1%)		72 (59.5)	70 (57.8)	
Ischemic cardiomyopathy	64 (42.4%)	63 (42.9%)		49 (40.5)	51 (42.2)	

Serum creatinine was significantly higher in the metoprolol succinate group than in the amiodarone group (79.34 ± 6.53 vs. 77.42 ± 5.81, *P* = 0.016) (Table 1). There were no differences between the two groups in relation to sex (male, 52.1 vs. 51.2%, *P* = 0.898), age (62.4 ± 4.7 vs. 61.2 ± 5.4 years, *P* = 0.066), diabetes (30.6 vs. 33.9%, *P* = 0.582), hypertension (58.7 vs. 61.2%, *P* = 0.694), smoking (34.7 vs. 36.4%, *P* = 0.788), digoxin (94.2 vs. 92.6%, *P* = 0.605), furosemide (all 100%), spironolactone (all 100%), dosage of perindopril (12.3 ± 2.2 vs. 12.4 ± 2.4 mg, *P* = 0.787), diameter of the left atrium (534 ± 2.1 vs. 53.7 ± 2.4 mm, *P* = 0.543), duration of AF (5.3 ± 2.7 vs. 5.1 ± 2.5 month, *P* = 0.438), and types of cardiopathy (*P* = 0.794) (Table 1).

The median follow-up time was 323 (95%CI: 192–368) days, calculated by the Reverse Kaplan-Meier method. Cardiovascular mortality (4.9 vs. 12.4%, HR: 2.500, 95%CI: 1.002–6.237, *P* = 0.040) and the first hospitalization for HF rate (52.9 vs. 67.8%, HR: 1.281, 95%CI: 1.033–1.589, *P* = 0.024) were significantly lower in the amiodarone group than in the metoprolol succinate group (Table 2). The average ventricular rate in the amiodarone group was significantly lower than in the metoprolol succinate group (64.5 ± 3.2 vs. 72.4 ± 4.2, *P* < 0.001). After 1 year of follow-up, the rate of sinus rhythm was significantly higher in the amiodarone group (*n* = 47, 38.8%) than in the metoprolol succinate group (*n* = 9, 7.4%) (HR: 0.191, 95%CI: 0.098–0.374, *P* < 0.001) (Table 2).

**Table 2:** Cardiovascular mortality, the first hospitalization for HF, and incidence of sinus rhythm conversion after 1-year follow-up.

	**Amiodarone (*n* = 121)**	**Metoprolol succinate (*n* = 121)**	**HR (95%CI)**	***P***
Cardiovascular mortality, *n* (%)	6 (4.9)	15 (12.4)	2.500 (1.002–6.237)	0.040
Rate of sinus rhythm conversion after a 1-year follow-up, *n* (%)	47 (38.8)	9 (7.4)	0.191 (0.098–0.374)	< 0.001
The first hospitalization for HF rate, *n* (%)	64 (52.9)	82 (67.8)	1.281 (1.003–1.589)	0.024
Mean ventricular rate	64.5 ± 3.2	72.4 ± 4.2		< 0.001

The difference in proBNP before and after treatment was significantly higher in the amiodarone group compared to the metoprolol group (3,914.88 vs. 2,558.07, *P* < 0.001). Meanwhile, the difference in LVEF was significantly higher in the amiodarone group than in the metoprolol group (−6.89 vs. −0.98, *P* < 0.001) (Table 3).

**Table 3:** Comparison of proBNP and LVEF between two groups.

	**Amiodarone (*n* = 121)**	**Metoprolol succinate (*n* = 121)**	***P***
**proBNP (pg/ml)**			
Before treatment	6452.30 ± 1124.45	6236.57 ± 1232.71	
After treatment	2537.42 ± 876.52	3678.50 ± 1121.64	
Difference	3914.88	2558.07	< 0.001
*P*	< 0.001	< 0.001	
**LVEF (%)**			
Before treatment	32.59 ± 3.49	33.23 ± 3.38	
After treatment	39.48 ± 2.17	34.21 ± 1.40	
Difference	−6.89	−0.98	< 0.001
*P*	< 0.001	< 0.001	

proBNP, NT-proB-type natriuretic peptide; LVEF, left ventricular ejection fraction.

The logistic regression results showed that serum creatinine (OR = 1.04, 95%CI: 0.97–1.12, *P* = 0.292), duration of AF (OR = 0.94, 95%CI: 0.78–1.13, *P* = 0.515), and type of cardiomyopathy (OR = 1.57, 95%CI: 0.63–3.90, *P* = 0.330) were not associated with cardiovascular mortality (Table 4). The logistic regression results showed that serum creatinine (OR = 1.00, 95%CI: 0.96–1.04, *P* = 0.952), duration of AF (OR = 0.97, 95%CI: 0.87–1.07, *P* = 0.493), and type of cardiomyopathy (OR = 1.15, 95%CI: 0.69–1.95, *P* = 0.590) were not associated with the first hospitalization for HF rate (Table 4).

**Table 4:** Logistic regression analysis for cardiovascular mortality and the first hospitalization for HF.

	**OR**	**95%CI**	***P***
**Cardiovascular mortality**			
Serum creatinine	1.041	0.966–1.121	0.292
Duration of AF	0.941	0.782–1.131	0.515
Types of cardiomyopathy	1.571	0.633–3.902	0.330
**First hospitalization for HF**			
Serum creatinine	1.001	0.960–1.044	0.952
Duration of AF	0.965	0.870–1.069	0.493
Types of cardiomyopathy	1.154	0.685–1.945	0.590

## Discussion

As previously reported, β-receptor blockers and amiodarone can be used to control ventricular rate in patients with HFrEF and pAF with RVR (13); however, amiodarone might have a better ability to restore and maintain sinus rhythm in patients with HFrEF and AF than β-receptor blockers (14, 17, 18). In this prospective observational study, the amiodarone group had lower risk of cardiovascular death and the first hospitalization for HF than metoprolol in HFrEF and persistent atrial fibrillation (pAF) with RVR. The mechanism may be related to improved cardiac function, rhythm control and ventricular rate control. These results highlight the clinical significance of using amiodarone in patients with HFrEF complicated with pAF and RVR. Through observing the patient's response to amiodarone, such as EF improvement, may help guide subsequent treatment strategies in patients with HFrEF complicated with pAF and RVR.

HF complicated with AF share many similar etiologies and risk factors, and both can induce and aggravate each other through mechanisms such as cardiac remodeling, neuroendocrine activation, and cardiac function decline caused by arrhythmias (23, 24). Previous studies recommended β-receptor blockers for patients with HF and AF (5); however, it was found that β-receptor blockers do not reduce all-cause mortality and cardiovascular mortality in patients with HF and AF (25–27).

Current guidelines recommend radiofrequency ablation to achieve cardioversion in patients with HFrEF complicated with pAF with RVR; yet, patients who do not qualify for non-pharmacological rate control, i.e., atrioventricular node ablation and pacing, can use amiodarone as a last resort (12). In addition, amiodarone can also be used as an atrioventricular nodal blocking agent for rate control (12), while intravenous amiodarone might be considered for acute rate control in critically ill or severely depressed LVEF patients (12). Nevertheless, additional studies are necessary to examine the clinical benefits of oral amiodarone in patients with HFrEF complicated with pAF with RVR.

The previous AATAC study (28) suggested that catheter ablation was superior to amiodarone in controlling AF at long-term follow-up and reducing unplanned hospitalization and mortality in patients with HF and persistent AF. However, the limitations of that study were that it neglected the patients with heart function grade IV, the proportion of patients with dilated cardiomyopathy was low, the average left atrial diameter was < 50 mm, and the application of CRT-D and the improvement of mitral regurgitation might help reduce the occurrence of AF. Therefore, the reported results did not represent all patients with HF and AF. Still, all the selected patients in our study were cardiac function grade IV after admission, had AF with an average duration of about 5 months, and had an average age >60 years. They were combined with ischemic cardiomyopathy or dilated cardiomyopathy, and the average left atrial diameter was >50 mm.

Regarding the choice of treatment for patients in the amiodarone group after the follow-up, if the patients in the amiodarone group recovered sinus rhythm at the end of the follow-up and the ejection fraction was significantly improved, we recommended continuing active sinus rhythms maintenance strategies such as catheters ablation or continued amiodarone to maintain sinus rhythm. On the other hand, if the ejection fraction of the patients in the amiodarone group did not significantly improve at the end of follow-up or the patients later developed symptomatic bradycardia, and amiodarone-related side effects, treatment of HF based on ventricular rate control was considered.

Our results showed that serum NT-proBNP in patients from the amiodarone group was significantly decreased compared to the application of metoprolol succinate, and the LVEF in patients with the amiodarone group was significantly increased compared to the application of metoprolol succinate, which indicated that the patient's heart function might be improved. Furthermore, the proportion of turn to a sinus rhythm and the average ventricular rate in the amiodarone group was more controlled, indicating that maintaining sinus rhythm and lower ventricular rate may facilitate improvement of the patient's heart function. In addition, cardiovascular mortality and the first hospitalization for HF were lower in the amiodarone group than in the metoprolol group, indicating that amiodarone may reduce the risk of cardiovascular death and the rate of the first hospitalization for HF.

This study has some limitations. First, the lack of randomization was a bias in this study. There may also be selection bias and information bias in this study. Second, only limited biomarkers were assessed. The heart structural changes and limited observational index were not assessed either. Third, the follow-up time of the study was short, and the prognosis of patients after 1 year was not evaluated, which may have a certain impact on the research results. Fourth, perindopril was used instead of sacubitril valsartan, which is known to significantly reduce the risks of death and hospitalization for HF in patients with HFrEF. Fifth, the study design prevented the inference of any causal relationship. Lastly, only Chinese patients with HFrEF complicated with pAF with RVR were included, and the findings cannot be generalized to patients in other countries. Therefore, controlled trials with larger sample sizes at multiple centers are needed to provide more medication experience and evidence of the application of oral amiodarone for the treatment of patients with HFrEF complicated with pAF with RVR in the future.

The high rates of loss to follow-up during follow-up in observational studies can bias the results. Patients who participated in the observational study were lost to follow-up mainly because they were living in rural areas, had adverse effects and self-stopped medication, or could not adhere to follow-up due to age, inconvenient transportation, being unaccompanied to the examinations by family members, decreased activity tolerance, and other reasons. In order to avoid the loss of follow-up bias caused by these reasons, the attending physician communicated with the family members when signing the informed consent and emphasized the importance of follow-up to ensure prognosis and supervise the patients to attend the follow-up on time.

Although the regression analysis could control confounding factors, there are still some factors missing or not recorded, such as the frequency of hospitalization before enrollment, which may also cause certain selection and information bias. For example, patients in the amiodarone group tended to have a higher frequency of hospitalization and more severe symptoms before enrollment. The advantages is patients are easy to accept amiodarone treatment with high compliance, but the shortage is the possibility of selection bias. Therefore, the protective effect of amiodarone treatment on HFrEF complicated with pAF with RVR may still be underestimated, and its real effect needs to be further studied.

In conclusion, in this prospective observational study, the amiodarone group had lower risk of cardiovascular death and the first hospitalization for HF than metoprolol in HFrEF and persistent atrial fibrillation (pAF) with RVR. The mechanism may be related to improved cardiac function, rhythm control and ventricular rate control.

## Data availability statement

The datasets presented in this study can be found in online repositories. The names of the repository/repositories and accession number(s) can be found in the article/supplementary material.

## Ethics statement

The studies involving human participants were reviewed and approved by the Institutional Ethics Board of the People's Hospital of Chongqing Hechuan. The patients/participants provided their written informed consent to participate in this study. The animal study was reviewed and approved by the Institutional Ethics Board of the People's Hospital of Chongqing Hechuan. Written informed consent was obtained from the individual(s) for the publication of any potentially identifiable images or data included in this article.

## Author contributions

YL: study design and data acquisition. YH: financial support, data analysis, and technical support. Both authors contributed to writing, revising, and approving the manuscript.
